# Dendritic Cells Actively Limit Interleukin-10 Production Under Inflammatory Conditions *via* DC-SCRIPT and Dual-Specificity Phosphatase 4

**DOI:** 10.3389/fimmu.2018.01420

**Published:** 2018-06-22

**Authors:** Jonas Nørskov Søndergaard, Simon J. van Heeringen, Maaike W. G. Looman, Chunling Tang, Vassilis Triantis, Pauline Louche, Eva M. Janssen-Megens, Anieta M. Sieuwerts, John W. M. Martens, Colin Logie, Hendrik G. Stunnenberg, Marleen Ansems, Gosse J. Adema

**Affiliations:** ^1^Radiotherapy & OncoImmunology Laboratory, Department of Radiation Oncology, Radboud Institute for Molecular Life Sciences, Radboud University Medical Center, Nijmegen, Netherlands; ^2^Department of Molecular Developmental Biology, Faculty of Science, Radboud Institute for Molecular Life Sciences, Radboud University, Nijmegen, Netherlands; ^3^Department of Molecular Biology, Faculties of Science and Medicine, Radboud Institute for Molecular Life Sciences, Radboud University, Nijmegen, Netherlands; ^4^Department of Medical Oncology and Cancer Genomics Netherlands, Erasmus MC Cancer Institute, Erasmus University Medical Center, Rotterdam, Netherlands

**Keywords:** ZNF366, ERK, chromatin immunoprecipitation sequencing, dual-specificity phosphatase 4, dendritic cells, MAPK, DC-SCRIPT, IL-10

## Abstract

Dendritic cell (DC)-based immunotherapy makes use of the DC’s ability to direct the adaptive immune response toward activation or inhibition. DCs perform this immune orchestration in part by secretion of selected cytokines. The most potent anti-inflammatory cytokine interleukin-10 (IL-10) is under tight regulation, as it needs to be predominantly expressed during the resolution phase of the immune response. Currently it is not clear whether there is active suppression of IL-10 by DCs at the initial pro-inflammatory stage of the immune response. Previously, knockdown of the DC-specific transcription factor DC-SCRIPT has been demonstrated to mediate an extensive increase in IL-10 production upon encounter with pro-inflammatory immune stimuli. Here, we explored how DC-SCRIPT contributes to IL-10 suppression under pro-inflammatory conditions by applying chromatin immunoprecipitation sequencing analysis of DC-SCRIPT and the epigenetic marks H3K4me3 and H3K27ac in human DCs. The data showed binding of DC-SCRIPT to a GA-rich motif at H3K27ac-marked genomic enhancers that associated with genes encoding MAPK dual-specificity phosphatases (DUSPs). Functional studies revealed that upon knockdown of DC-SCRIPT, human DCs express much less DUSP4 and exhibit increased phosphorylation of the three major MAPKs (ERK, JNK, and p38). Enhanced ERK signaling in DC-SCRIPT-knockdown-DCs led to higher production of IL-10, which was reverted by rescuing DUSP4 expression. Finally, DC-SCRIPT-knockdown-DCs induced less IFN-γ and increased IL-10 production in naïve T cells, indicative for a more anti-inflammatory phenotype. In conclusion, we have delineated a new mechanism by which DC-SCRIPT allows DCs to limit IL-10 production under inflammatory conditions and potentiate pro-inflammatory Th1 responses. These insights may be exploited to improve DC-based immunotherapies.

## Introduction

Immunotherapy has gained great success in recent years due to the success of checkpoint inhibitors targeting the PD-1/PD-L1 or CTLA-4 pathways (1–4). These checkpoint inhibitors act on T cells, and it is widely accepted that T cells are quintessential in the fight against cancer. However, T cells are not capable of eliciting an anti-tumor response by themselves, because without the right instructions, the T cells will remain naïve or become tolerogenic (5). The only cell type capable of efficiently educating naïve T cells is the dendritic cell (DC). We have come very far in our understanding of the role of cell surface receptors and secreted molecules involved in immune regulation by DCs, which has been exploited in therapies against cancer with promising results (6, 7). By contrast, information regarding intracellular regulatory circuits in DCs is far scarcer, and could possess potential for future therapeutic strategies as well.

DCs sample their environment, and sense foreign molecules using pattern-recognition receptors of which the toll-like receptors (TLRs) are the best known (8). Activation of DCs through TLR-triggering is needed for upregulation of antigen-presenting and co-stimulatory molecules, and production of cytokines that direct the T cell response toward activation (9). Thus, DCs hold the potential to induce either an immune-activating or immune-dampening response (10). Interleukin-10 (IL-10) has been demonstrated to play a crucial role in this process, by inhibiting development of pro-inflammatory Th1 cells and promoting development of anti-inflammatory Tr1 cells (11). Besides being involved in T cell education, several studies also reported on downregulation of MHC class II expression on antigen-presenting cells (APCs) and of MHC class I on tumor cells in cancer patients with elevated IL-10 levels in serum or tumors (12–18). Although high IL-10 production in cancer patients is associated with a poor prognosis, some recent reports have suggested that situations may exist in which elevated levels of IL-10 in cancer patients may also have beneficial effects *via* dampening the chronic inflammation in tumor microenvironments and by stimulation of cytotoxicity of already activated CD8+ T cells (19).

In the context of APCs, we have learned a great deal about how IL-10 is upregulated *via* activation of the MAP kinase ERK and the transcription factor NF-κB during the resolution phase of an immune response (20). Whether IL-10 expression is actively kept in check in DCs during the initial inflammatory phase of the immune response is currently unknown (20). Dendritic cell-specific transcript (DC-SCRIPT, *ZNF366*) is a transcription factor uniquely expressed by DCs in the immune system (21–23). DC-SCRIPT has been shown to have a complex collection of functions, including nuclear receptor co-regulatory activity (24–29), cell cycle regulation (30), NF-κB activity modulation (31), and mediating induction of T cell proliferation and IFN-γ production (23). Most strikingly, DC-SCRIPT knockdown leads to a massive increase in IL-10 production after pro-inflammatory TLR-triggering (23, 27, 31), suggesting that DC-SCRIPT may participate in active suppression of IL-10. In this study. we explored how DC-SCRIPT contributes to the active suppression of IL-10 production in human activated monocyte-derived DCs.

## Results

### DC-SCRIPT Binds Genomic Enhancers Near MAPK Phosphatases

**Figure 1:**
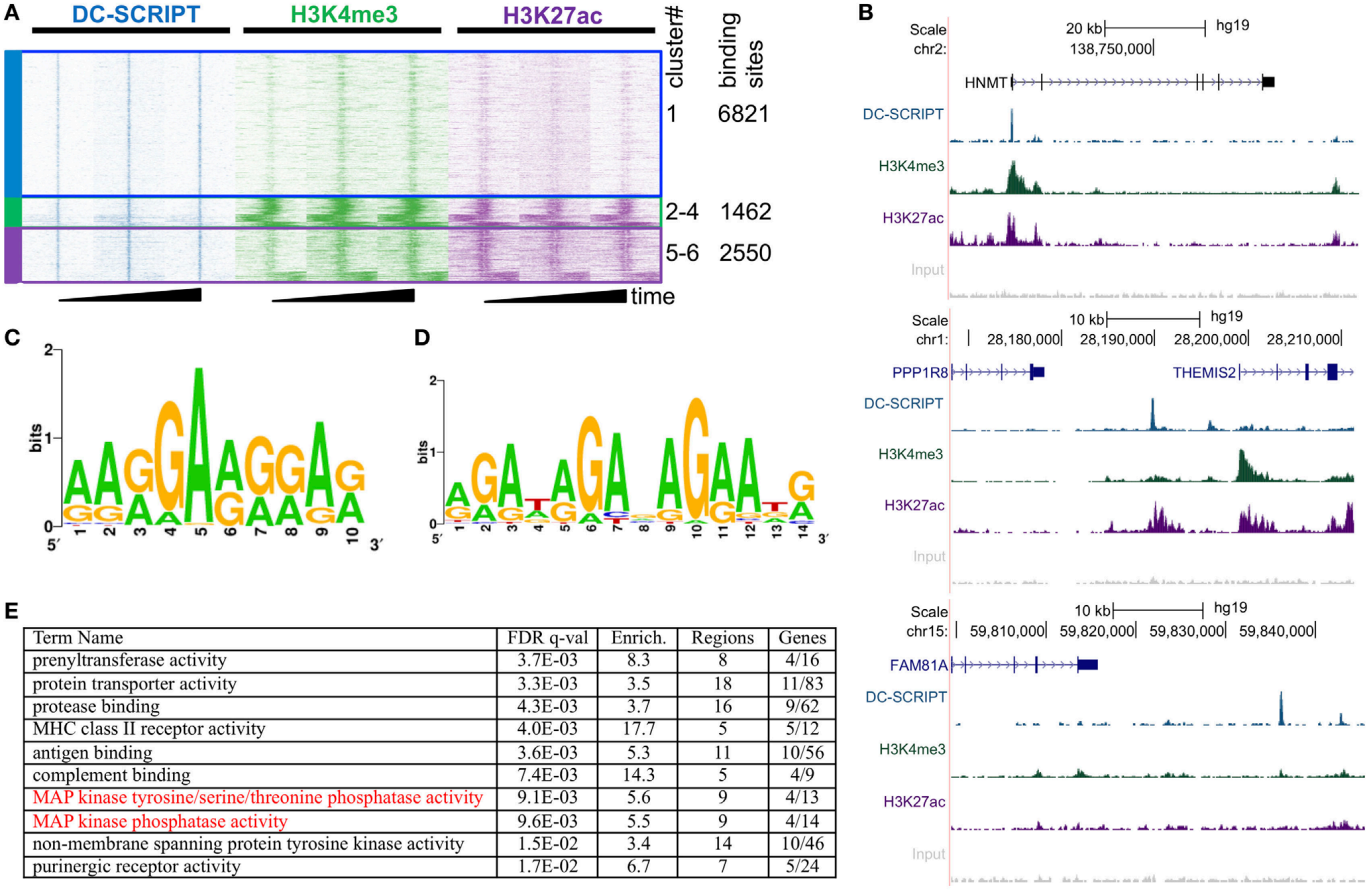
Genome-wide mapping of DC-SCRIPT binding sites in human dendritic cells (DCs). Chromatin immunoprecipitation sequencing (ChIP-Seq) of human immature or R848 (toll-like receptors 7/8 ligand)-activated DCs using DC-SCRIPT, H3K4me3, or H3K27ac Abs (*n* = 3). **(A)** Heatmap of *k*-means clustering analysis (*k* = 6, Euclidean distance) of DC-SCRIPT (blue), H3K4me3 (green), and H3K27ac (purple) in 10-kb windows around DC-SCRIPT peak summits. Clusters 2–4 are merged into an enhancer-associated cluster; clusters 5–6 are merged into a promoter-associated cluster. The rows correspond to the peaks; the *x*-axis shows the position relative to the peak center. The intensity of the color represents the number of reads in 100-bp windows. **(B)** Representative UCSC Genome Browser tracks of a PA-SC binding site (top), an EA-SC binding site (middle), and a no mark binding site (bottom). The tracks from top to bottom show the gene annotation, the ChIP-Seq signal for DC-SCRIPT (blue), H3K4me3 (green), H3K27ac (purple), and input (gray). **(C)** *de novo* motif generated from DC-SCRIPT ChIP-Seq binding sites. **(D)** *In vitro* motif identified by cyclic amplification and selection of targets. **(E)** Genomic Regions Enrichment of Annotations Tool analysis of all DC-SCRIPT-binding sites containing the GA-rich motif. The table contains the top 10 most significant terms in the gene ontology category molecular function. See also Figure S1 and Tables S1 and S2 in Supplementary Material.

In order to delineate the molecular mechanism for how DC-SCRIPT regulates IL-10 expression in DCs under inflammatory conditions, we conducted a chromatin immunoprecipitation sequencing (ChIP-Seq) analysis using a DC-SCRIPT specific antibody on human monocyte-derived DCs. Antibodies specific for the epigenetic marks histone H3 lysine 4 tri-methylation [H3K4me3; associated with promoters of active genes (32)] and H3K27 acetylation (ac) [present at active enhancers and promoters (33)] were taken along in the same DC samples (Figure 1; GEO accession number: GSE78923). To account for donor variation and dynamics, three donors were assayed, using immature, 1 h, and 24 h TLR ligand R848 (Resiquimod)-stimulated DCs. Only DC-SCRIPT binding sites consistently present in all three donors in at least one of the assayed time points were used for subsequent analysis, yielding a total of 10,833 DC-SCRIPT binding sites in the human genome. Clustering of the DC-SCRIPT DNA binding sites with the genomic H3K27ac and H3K4me3 histone marks in DCs displayed a 37% overlap (Figure 1A, cluster 2–6). Out of these, 1,462 DC-SCRIPT binding sites overlapped with the promoter mark H3K4me3 (Figure 1A, clusters 2–4 and Figure 1B, *top*), and 2,550 DC-SCRIPT binding sites overlapped with histone marks characteristic for enhancers (high H3K27ac, low/no H3K4me3, clusters 5–6 Figures 1A,B, *middle*). Promoter-associated DC-SCRIPT binding sites are henceforth referred to as PA-SC binding sites and enhancer-associated DC-SCRIPT binding sites referred to as EA-SC binding sites. The DC-SCRIPT binding sites that did not co-localize with either of these marks (Figures 1A,B, *bottom*) were left out from the current analysis. To further characterize the sequence content of the genomic locations where DC-SCRIPT binds, a *de novo* motif analysis was performed on the DC-SCRIPT ChIP-Seq dataset using GimmeMotifs (34). This unguided comparison of the DNA sequence under all the DC-SCRIPT binding sites, yielded a GA-rich DC-SCRIPT binding motif (Figure 1C). To validate the motif in a different and independent assay, we also performed *in vitro* cyclic amplification and selection of targets (CAST) in a cell-free system (Figure 1D). The CAST motif successfully validated the *de novo* motif with the two independently generated motifs being 78% similar to each other [assayed by MAST in MEME (35), Figure S1 in Supplementary Material]. The *de novo* and the *in vitro* motif were found in up to 44% of the EA-SC binding sites and 38% of the PA-SC binding sites, respectively (Figure S1 in Supplementary Material).

Previously, we have shown that in the absence of DC-SCRIPT there is increased NF-κB binding to the *il10* enhancer (31), suggesting that DC-SCRIPT may also bind there. Surprisingly, there was no DC-SCRIPT binding site in the *il10* promoter or any *il10*-associated enhancer that could explain the effect of DC-SCRIPT expression on IL-10 production. Genomic Regions Enrichment of Annotations Tool [GREAT (36)] was then used to get more insight into the pathways that DC-SCRIPT may regulate to affect IL-10 expression. The gene ontology (GO) biological process term was dominated by immune regulatory processes (Table S1 in Supplementary Material), reinforcing the previously demonstrated role of DC-SCRIPT in immune regulation (23, 31). Interestingly, the molecular function GO showed that DC-SCRIPT binds in the vicinity of MAPK phosphatase genes (Figure 1E; Table S2 in Supplementary Material). MAPK phosphatases are a subgroup of the dual-specificity phosphatases (DUSPs) and are responsible for the dephosphorylation of MAPKs (37). Given that the MAPK ERK previously has been associated with IL-10 production (20), DC-SCRIPT may therefore potentially regulate DUSPs to modulate IL-10 production.

### DC-SCRIPT Modulates the MAPK Pathway

**Figure 2:**
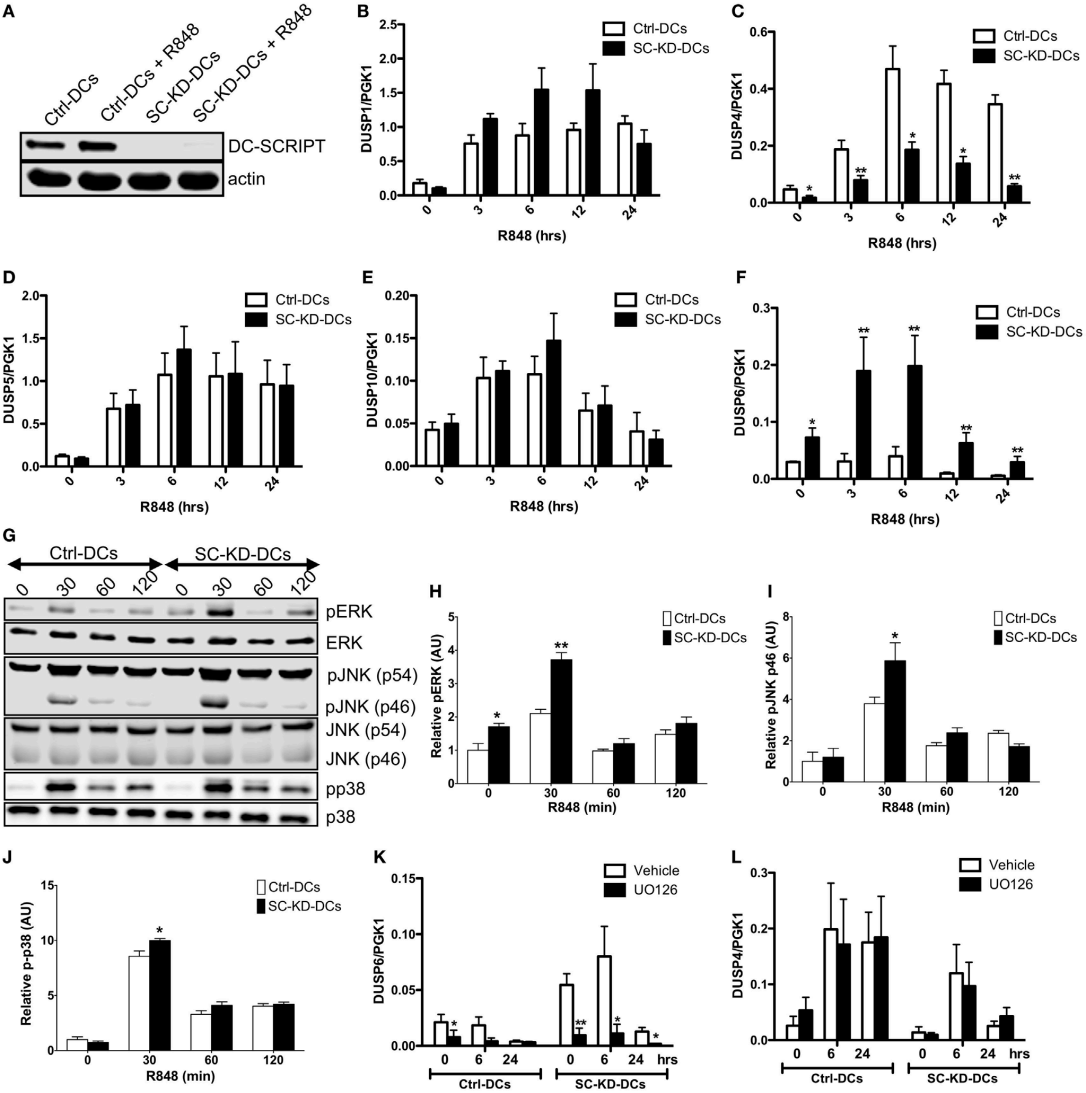
DC-SCRIPT affects the MAPK pathway. **(A)** Representative western blot of siRNA-mediated DC-SCRIPT knockdown in immature and R848-stimulated dendritic cells (DCs). **(B–F)** R848-stimulated Ctrl-DC and SC-KD-DC time course for dual-specificity phosphatase (DUSP)1, 4, 5, 6, and 10 (*n* = 4, mean + SEM). **(G–J)** Phosphorylation and total protein expression of ERK, JNK, and p38 in Ctrl-DCs and SC-KD-DCs after stimulation with R848 (*n* = 5, mean + SEM). **(K,L)** DUSP6 and DUSP4 expression in Ctrl-DCs and SC-KD-DCs pretreated with UO126 (MEK inhibitor) or vehicle control for 1 h, followed by R848 stimulation (*n* = 4, mean + SEM). Statistics: Student’s paired *t* test, Ctrl-DCs were compared to SC-KD-DCs at the same time point. *p < 0.05, **p < 0.01. See also Figure S2 in Supplementary Material.

The GREAT analysis showed that 4 out of 14 MAPK DUSPs were associated with a total of 9 EA-SC binding sites located on average 246 kb (range: 3–859 kb) from the transcription start site. In order to validate their expression in relation to DC-SCRIPT, we knocked down DC-SCRIPT using siRNA specific to DC-SCRIPT (SC-KD-DCs) or control non-targeting siRNA (Ctrl-DCs) (Figure 2A). The expression of the four DUSP genes that had DC-SCRIPT binding sites associated (DUSP1, DUSP4, DUSP5, and DUSP10) were assayed by RT-qPCR at different time points after R848 stimulation (Figures 2B–E). As a control, the well-known ERK phosphatase DUSP6 (38), that in this study did not have any DC-SCRIPT binding sites associated, was also assayed (Figure 2F). All five assayed DUSPs were upregulated after R848 stimulation, but only DUSP4 and DUSP6 displayed a significant difference in expression upon DC-SCRIPT knockdown. DUSP4 was consistently higher expressed in Ctrl-DCs, while DUSP6 was consistently higher expressed in SC-KD-DCs. Since the main role of DUSPs is to inhibit MAPK signaling, we next assayed if DC-SCRIPT silencing has an impact on the signaling of the MAPKs. The levels of phosphorylation of the three major MAPKs: ERK, JNK, and p38 were assayed by western blotting (WB) (Figures 2G–J) and flow cytometry (Figure S2 in Supplementary Material). Stimulation with R848 led to a peak in phosphorylation of all three kinases after 30 min in both SC-KD-DCs and Ctrl-DCs. DC-SCRIPT knockdown significantly enhanced the phosphorylation of all three kinases, with the most potent increase in ERK, and only a minor change of phosphorylation on p38. As ERK signaling has previously been demonstrated to upregulate expression of DUSPs (39, 40), we pretreated the cells with a MEK inhibitor prior to stimulation, to investigate whether ERK signaling was responsible for the observed effect on DUSP4 and DUSP6 expression (Figures 2K,L). Strikingly, pre-treatment with the inhibitor diminished DUSP6 upregulation, while DUSP4 remained unaltered. This suggests that DUSP4 is regulated directly by DC-SCRIPT, whereas the effect on DUSP6 is rather part of an ERK signaling feedback loop.

### DC-SCRIPT Enhances DUSP4 Expression

**Figure 3:**
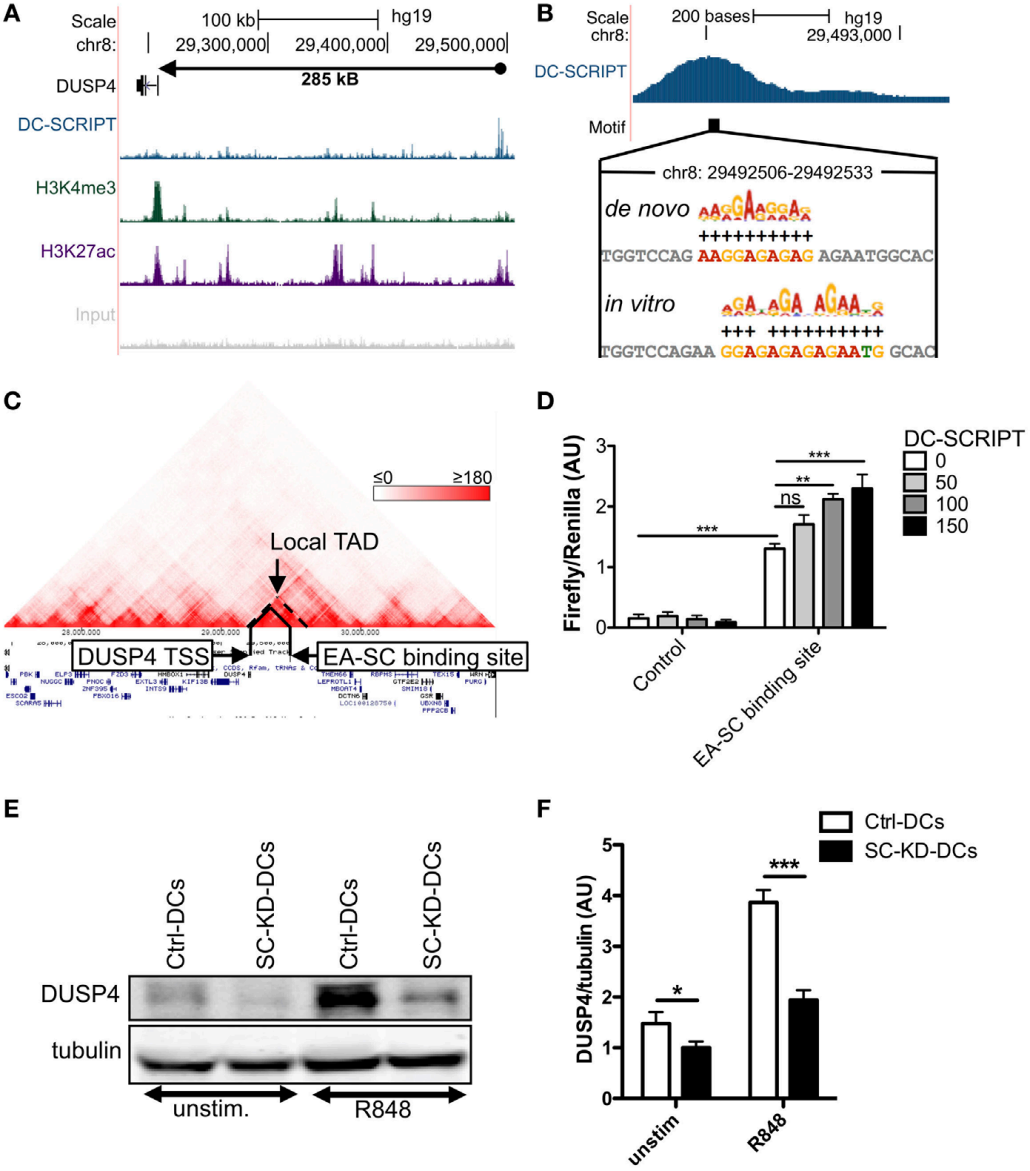
DC-SCRIPT enhances dual-specificity phosphatase (DUSP)4 expression. **(A,B)** DUSP4 EA-SC binding site track from UCSC Genome Browser. **(A)** The tracks from top to bottom show the DUSP4 gene annotation, and the ChIP-Seq signal for DC-SCRIPT (blue), H3K4me3 (green), H3K27ac (purple), and input (gray). **(B)** Position of the GA-rich motifs identified *de novo* from this ChIP-Seq dataset or *in vitro* by cyclic amplification and selection of targets. **(C)** Topological associated domain (TAD) analysis was done using the Hi-C data browser from the Yue-lab at Penn State (http://promoter.bx.psu.edu/hi-c) containing data from Ref. (41–43). Highlighted in the figure is a local TAD that only contains the DUSP4 gene, and the EA-SC binding site. The example shown is from GM12878 (lymphoblastoid B cell line). **(D)** Luciferase assays with DUSP4 promoter-containing vector with either genomic DNA containing the DUSP4 gene-associated EA-SC binding site, or a control piece of DNA located in between the binding site and the DUSP4 TSS of similar length. Some conditions were cotransfected with increasing amounts of a DC-SCRIPT expression vector or corresponding amount of empty vector. Firefly luciferase is presented relative to TK renilla luciferase control (*n* = 3, mean + SEM). Statistics: ANOVA with Bonferroni post test. **(E,F)** Protein expression level of DUSP4 in unstimulated or 6 h R848-stimulated Ctrl-DCs and SC-KD-DCs (*n* = 7, mean + SEM). Statistics: Student’s paired *t* test. **p < 0.01, ***p < 0.001.

The DC-SCRIPT binding site associated with the DUSP4 gene was further validated by ChIP-qPCR in seven additional independent donors (Figure S3 in Supplementary Material). The binding site displayed enhancer characteristics (high H3K27ac and low H3K4me3 binding) and was located 285kB downstream of the DUSP4 transcription start site (Figure 3A). Both the *de novo* and the *in vitro* motif could be found toward the center of the binding site (Figure 3B). Intriguingly, this location falls within a local topological associated domain (TAD) previously found in all 19 different assayed cell lines and 13 different tissues, including immune cells (Figure 3C). Together this would suggest that this DC-SCRIPT binding position is an enhancer for DUSP4. To further evaluate this, a luciferase vector containing the DUSP4 promoter and the genomic DNA underlying the DUSP4 EA-SC-binding site was generated. For comparison, a control vector with a piece of genomic DNA of similar size, without the motif, and located between the DUSP4 TSS and the DUSP4 EA-SC binding site was employed. Interestingly, the genomic DNA underlying the DUSP4 EA-SC genomic DNA lead to a 8.5-fold increase in luciferase activity (Figure 3D). Co-transfection with DC-SCRIPT further increased the luciferase signal in a dose-dependent manner (1.8-fold compared to no DC-SCRIPT). To confirm the impact of DC-SCRIPT expression on DUSP4 at the functional level, DUSP4 protein expression was determined in immature and R848-stimulated Ctrl-DCs and SC-KD-DCs (Figures 3E,F). In accordance with the mRNA expression, DUSP4 protein levels were significantly higher in Ctrl-DCs relative to DC-SCRIPT silenced DCs both at the immature state and after stimulation. Altogether, these data therefore indicate that the DUSP4 gene-associated EA-SC binding site works as an enhancer, and that DC-SCRIPT expression impacts the DUSP4 protein level.

### The DC-SCRIPT-Induced Phenotype Is Mediated by DUSP4

**Figure 4:**
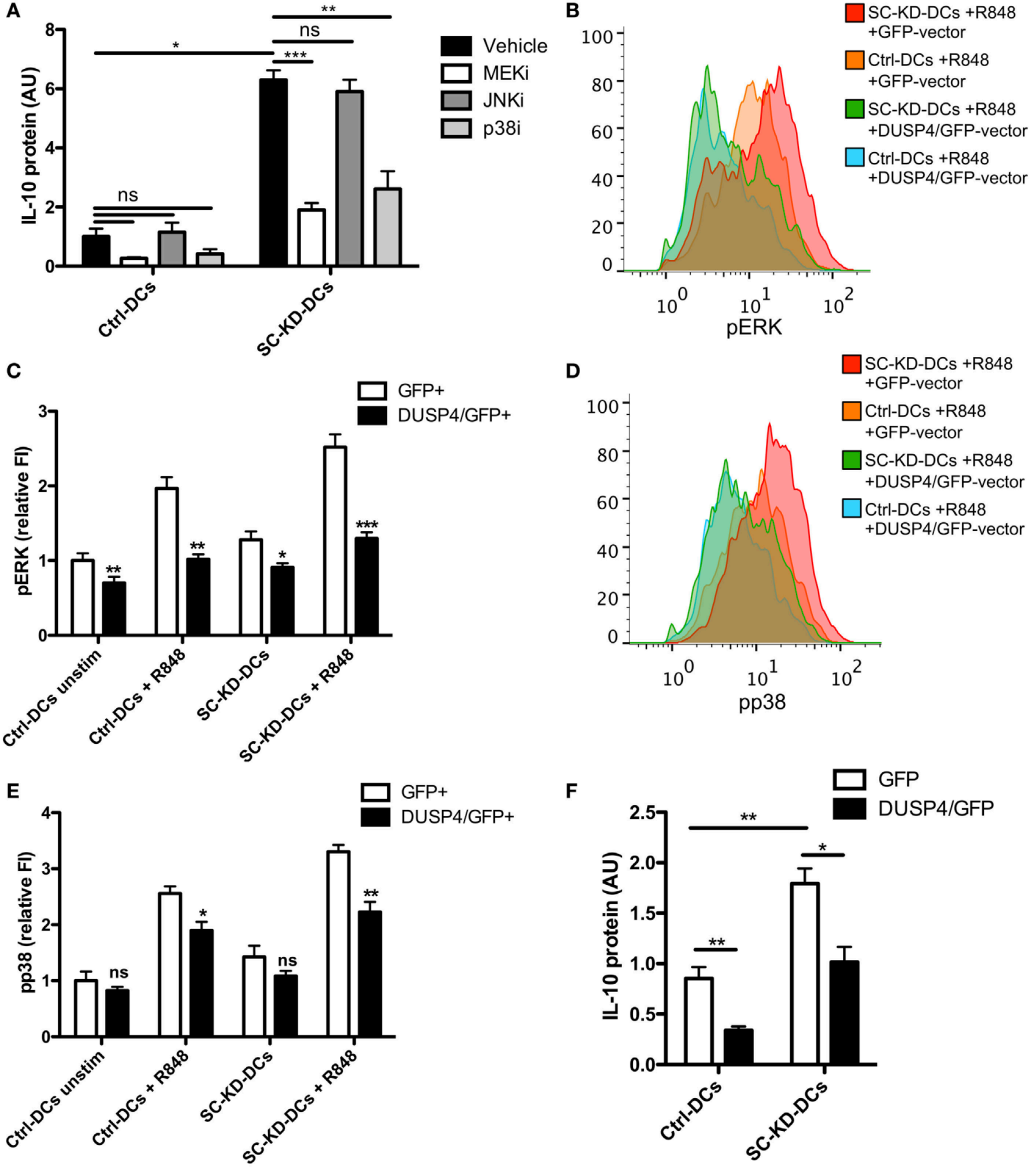
DUSP4 expression prevents the DC-SCRIPT-knockdown-mediated change in ERK phosphorylation and IL-10 production. **(A)** IL-10 protein production by Ctrl-DCs and SC-KD-DCs pre-treated with inhibitors for MEK (upstream of ERK), JNK, p38 or with vehicle control and stimulated with R848 (*n* = 6, mean + SEM). **(B–E)** pERK and pp38 phosphorylation levels of Ctrl-DCs and SC-KD-DCs after overexpression of GFP or DUSP4/GFP by phosphoflow (*n* = 8, mean + SEM). **(F)** IL-10 protein production by Ctrl-DCs and SC-KD-DCs after overexpression of GFP or DUSP4/GFP (*n* = 7, mean + SEM). The IL-10 protein data have been normalized to account for donor variation and displayed as arbitrary units (SC-KD-DCs + vehicle range: 0.5–4.6 ng/mL) Statistics: Student’s paired *t* test. Where no lines are shown, Ctrl-DCs were compared to SC-KD-DCs at the same time point. *p < 0.05, **p < 0.01, ***p < 0.001. FI, fluorescence intensity; AU, arbitrary unit. See also Figure S4 in Supplementary Material.

The most dominant immunological phenotype reported for DC-SCRIPT in DCs so far is the major increase in IL-10 production following siRNA-mediated knockdown of DC-SCRIPT (23, 31). To assess whether the observed change in DUSP4 expression level could be responsible for this effect on IL-10 production, we evaluated Ctrl-DCs and SC-KD-DCs ability to produce IL-10 after MAPK inhibition and DUSP4 overexpression (Figure 4). While inhibition of JNK did not affect IL-10 secretion, both inhibition of the upstream kinase of ERK (MEK) and p38 lead to a major decrease in IL-10 (Figure 4A).

DUSP4 has previously been demonstrated to have a high affinity for dephosphorylating ERK (44), and could therefore explain the enhanced ERK phosphorylation after DC-SCRIPT knockdown. To confirm this in human DCs, we rescued DUSP4 expression by introducing DUSP4/GFP or GFP in SC-KD-DCs using Ctrl-DCs as controls and determined the level of phosphorylated ERK (Figures 4B,C; Figure S4 in Supplementary Material). Strikingly, rescuing DUSP4 expression changed phosphorylation of ERK in the SC-KD-DCs back to wild-type levels. As DC-SCRIPT knockdown also had an effect on phosphorylation of p38, the effect of DUSP4 on p38 phosphorylation was also assayed under the same settings (Figures 4D,E). Interestingly, like ERK, phosphorylation of p38 was also reduced to wild-type levels after rescuing DUSP4 in DC-SCRIPT knockdown DCs. Finally, rescuing DUSP4 expression in SC-KD-DCs also normalized levels of IL-10 production (similar level as Ctrl-DC) (Figure 4F).

### DC-SCRIPT Expression in DCs Skews Naïve T Cells Toward Th1 Immune Activation

**Figure 5:**
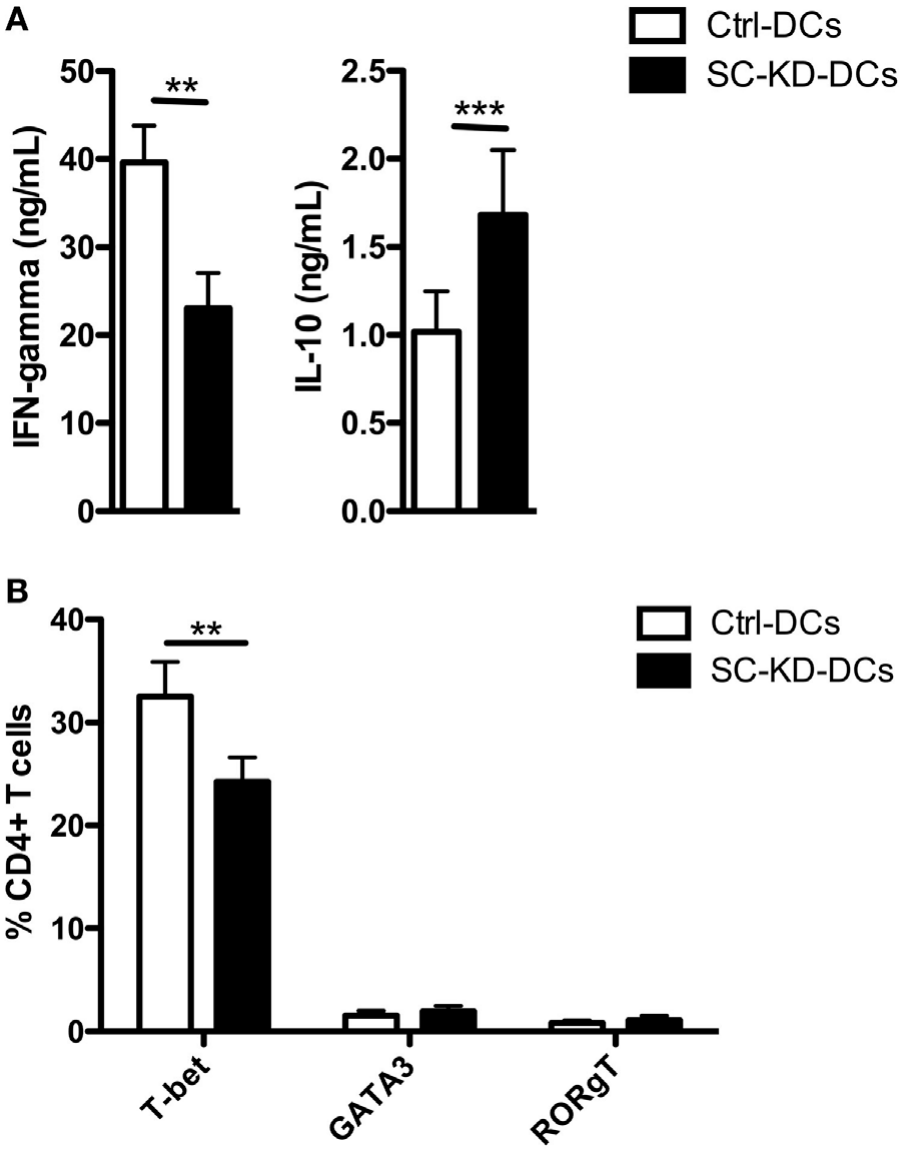
Knockdown of DC-SCRIPT in DCs polarizes T cells toward immunosuppression. Naïve CD4+ T cells were co-cultured with SC-KD-DCs or Ctrl-DCs until in a resting state after 10–12 days. Upon re-stimulation, the phenotype of the T cells were assayed for **(A)** expression of T cell subset markers by FACS and **(B)** secreted cytokines by ELISA (*n* = 12 + SEM). Statistics: Student’s paired *t* test. **p < 0.01, ***p < 0.001.

To determine the impact of DC-SCRIPT expression on the cytokine polarization of naïve CD4+ T cells, we co-cultured SC-KD-DCs or Ctrl-DCs with allogeneic naïve CD4+ T cells and assayed their cytokine production. Interestingly, the responding T cells produced less IFN-γ and more IL-10 upon incubation with SC-KD-DCs (Figure 5A). In line with these data, the expression of T cell subset restricted transcription factors revealed a significant decrease in T-bet (Th1 TF) expressing T cells, while the number of GATA3 (Th2 TF) and RORgT (Th17 TF) expressing T cells remained similar (Figure 5B).

**Figure 6:**
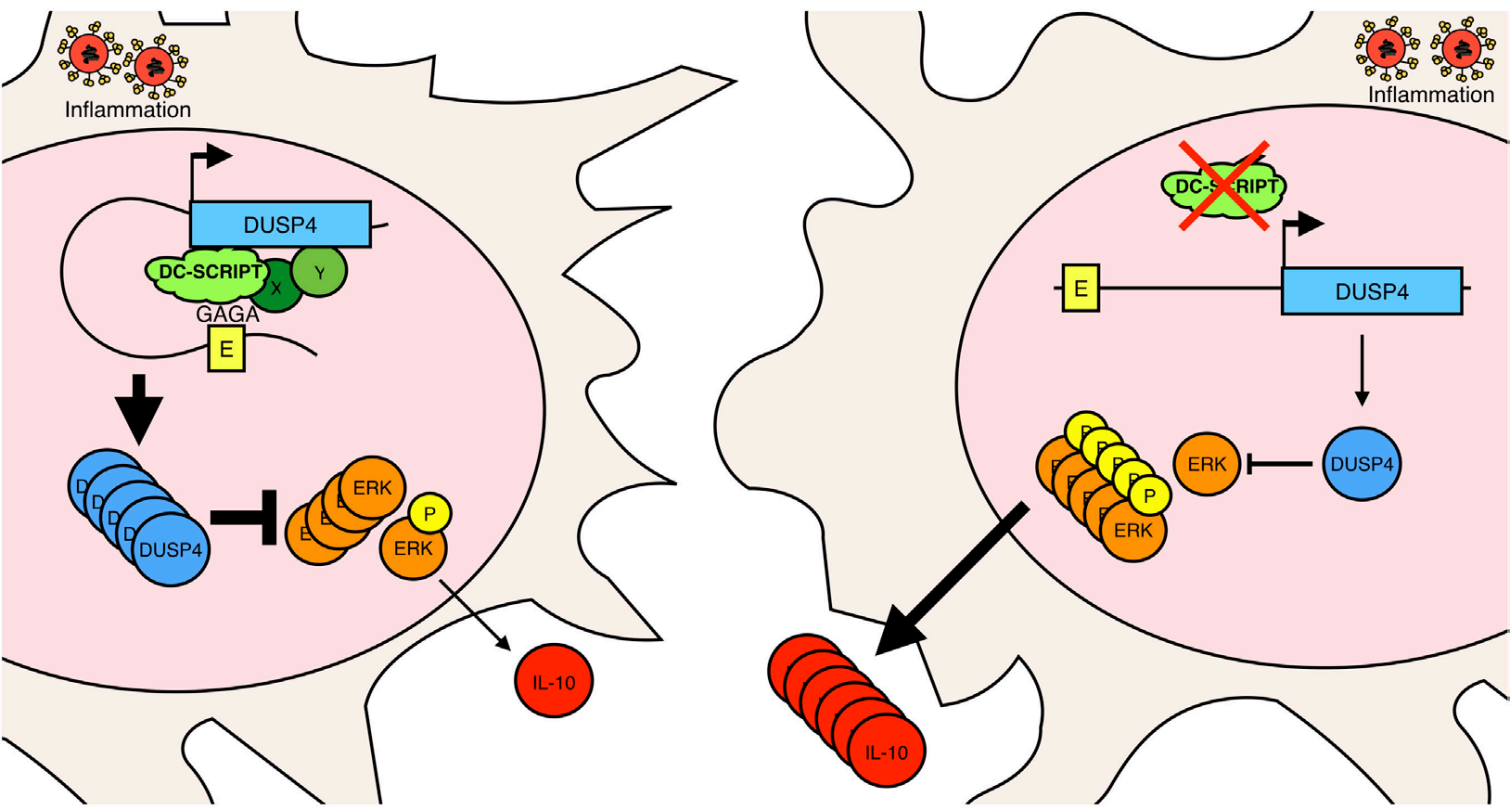
Model of DC-SCRIPT mediated control of IL-10 production in DCs. In the presence of DC-SCRIPT (left figure), DC-SCRIPT binds an enhancer (E) for DUSP4 *via* a GA-rich motif. This leads to enhanced DUSP4 expression, which limits ERK signaling, and subsequent IL-10 production under inflammatory conditions. By contrast, in the absence of DC-SCRIPT (right figure), DUSP4 is only expressed at a low level, thereby enabling higher ERK signaling leading to an increase in IL-10 production. X and Y are potential protein partners in the enhancer complex.

Altogether, these data demonstrate that DC-SCRIPT-mediated enhancement of DUSP4 expression is responsible for restricting ERK and p38 signaling and subsequent IL-10 production by professional antigen-presenting DCs under inflammatory conditions (Figure 6), which in turn limits pro-inflammatory CD4+ T cell polarization.

## Discussion

Dendritic cells are the sentinels of the immune system, playing a decisive role in the balance between immunogenic and tolerogenic immune responses (10), however, the molecular mechanism of how DCs control production of the anti-inflammatory cytokine IL-10 in an inflammatory setting is still unclear. Here, we show that DC-SCRIPT, a DC-specific transcription factor in the immune system, binds to GA-rich sequences in enhancer- and promoter-associated DNA regions for many immune-related genes, including an enhancer for the MAPK phosphatase DUSP4. Moreover, we show that DC-SCRIPT knockdown limits DUSP4 expression resulting in an increase in the activity of the MAPK signaling pathway and subsequent IL-10 production by DCs. In addition, the knockdown of DC-SCRIPT also hindered differentiation of naïve CD4+ T cells into pro-inflammatory T cells. Altogether, these data show a novel mechanism by which professional antigen-presenting DCs limit IL-10 production, a pathway that needs to be tightly controlled to induce protective immune responses (20).

Many different immune cells can produce IL-10 and during cancer development, this has mainly been considered detrimental for successful eradication of the tumor cells due to IL-10’s immunosuppressive capabilities on DCs (45) and T cells (46). Previously, we have demonstrated that DC-SCRIPT affects TLR-mediated IL-10 production in human DCs (23), and described that enhanced IL-10 production in SC-KD-DCs was partly caused by altered post-translational modifications of NF-κBp65 leading to increased binding of NF-κBp65 to an IL-10 enhancer element (31). In our current ChIP-Seq data, we did not find a DC-SCRIPT binding site at the same position as for NF-κBp65 in the enhancer of the IL-10 gene, i.e., it does not seem that DC-SCRIPT is part of the NF-κBp65 DNA binding complex. In line with these observations, knockdown of DC-SCRIPT affected the phosphorylation status of NF-κB (31), and with the current data linking DC-SCRIPT to phosphatases, it may suggest that DC-SCRIPT affects NF-κB activation indirectly. In support of this, a downstream kinase (MSK1) of the MAPKs ERK and p38 has been demonstrated to mediate phosphorylation of NF-κBp65 (47), and ERK has been shown to play a critical role for IL-10 production in DCs (20).

In the current work, we found that DC-SCRIPT knockdown leads to higher DUSP4 expression and that DC-SCRIPT binds a GA-rich DNA sequence with enhancer abilities within a local DUSP4-TAD. Using luciferase assays, we found that the largest enhancing effect was gained by cloning the enhancer close to the DUSP4 promoter, while co-transfecting DC-SCRIPT only led to a subtle (but significant) further increase in the signal. These data could indicate that one of the roles of DC-SCRIPT in enhancing DUSP4 expression would be to bring the DUSP4 enhancer in close proximity to the promoter. Currently, only little is known about the functional role of DUSP4 in DCs. Gene expression analysis has revealed that immune cells express up to 17 different DUSPs to various extent, but only one or two DUSPs showed high expression in any single cell type (37). DUSP4 was predominantly expressed in DCs, and furthermore, high DUSP4 expression could distinguish DCs from the closely related monocyte-derived macrophages (37). Interestingly, accumulating evidence suggests that cell type-specific transcription factors often regulate cell type-specific genes *via* binding to enhancers (48).

One of DCs main function in the immune system is to educate T cells toward pro-inflammatory or anti-inflammatory responses. We observed that knockdown of DC-SCRIPT in DCs limited the DCs capability to induce pro-inflammatory CD4+ T cells when polarized from naïve cells. These T cells produced less IFN-γ and more IL-10, and in line with these data, the number of T-bet expressing T cells was also reduced. Surprisingly, even though DC-SCRIPT knockdown leads to increased production of IL-10, and IL-10 is a potent inducer of anti-inflammatory Tr1 cells (11), preliminary experiments did not show any skewing of naïve T cells into Tr1 cells (data not shown). As DC-SCRIPT levels affect a DC’s capacity to induce inflammatory Th1 responses, one could consider monitoring DC-SCRIPT expression in DCs used in vaccination studies.

In summary, the transcription factor DC-SCRIPT binds regulatory DNA sequences linked to genes involved in the immune system and the MAPK pathway, including MAPK phosphatases. We identify regulation of expression of the MAPK phosphatase DUSP4 as the mode of action through which DC-SCRIPT restricts the expression of the crucial immune-inhibitory cytokine IL-10 in DCs. These data help to delineate the mechanisms that govern DCs unique molecular function, and its central role in controlling immune responses. As DC immunotherapy is a promising approach to treat cancer patients, specifically targeting tumor cells and having only few side effects (49, 50), much research has focused on which pattern-recognition receptor ligands or cytokine-cocktails would generate a DC-phenotype with a favorable cytokine profile, often focusing on high IL-12 and low IL-10 levels (51–53). The current data showing IL-10 being actively inhibited by DC-SCRIPT-induced phosphatases imply that maturing DCs with a combination of immune-activating adjuvants and either kinase inhibitors or phosphatase inducers may be beneficial to improve DC-based immunotherapy.

## Materials and Methods

### Generation of Human Monocyte-Derived Dendritic Cells (moDCs)

Human moDCs were generated from PBMCs as described previously (54). Buffy coats were obtained from healthy volunteers (Sanquin, Nijmegen, The Netherlands) after informed consent and according to institutional guidelines. Plastic-adhered monocytes were cultured for a total of 6 days in RPMI 1640 medium (Life Technologies) supplemented with 1% ultra-glutamine (Cambrex), 0.5% antibiotic–antimycotic (Invitrogen), 10% (v/v) FCS (Greiner Bio-one), IL-4 (300 U/mL), and GM-CSF (450 U/mL) (both from Cellgenix). On day 3, moDCs were supplemented with new IL-4 (300 U/mL) and GM-CSF (450 U/mL).

### Small Interfering RNA (siRNA)-Mediated Knockdown

On day 3–4 of DC differentiation, cells were harvested and subjected to electroporation. For DC-SCRIPT silencing, a 23-nt custom ZNF366 siRNA termed SC38 targeting the DC-SCRIPT gene at position 2,349–2,369 was used (Thermo Scientific). siRNA ON-TARGETplus Non-Targeting siRNA#1 (Thermo Scientific) was used as control. Cells were washed twice in PBS and once in OptiMEM without phenol red (Invitrogen). A total of 10 µg siRNA was transferred to a 4-mm cuvette (Bio-Rad), and 10 × 10^6^ DCs were added in 200 µL OptiMEM and incubated for 3 min before being pulsed with an exponential decay pulse at 300 V, 150 μF, in a Genepulser Xcell (Bio-Rad), as previously described (55). Immediately after electroporation, the cells were transferred to pre-heated (37°C) phenol red-free RPMI 1640 culture medium supplemented with 1% ultra-glutamine, 10% (v/v) FCS, IL-4 (300 U/mL), and GM-CSF (450 U/mL).

### Stimulations and Inhibitors

Immature DCs were stimulated with 4 µg/mL R848 (Axxora). In some experiments, DCs were pre-treated 1–2 h with one of the following inhibitors: 4 µM UO126 (MEK1/2, LC Laboratories), 2.5 µM SB203580 (p38, LC laboratories), 5 µM SP600125 (JNK, Tocris Bioscience).

### RNA Isolation, Reverse Transcription, and Quantitative PCR

Total RNA was isolated from cells using Trizol (Ambion). RNA quantity and purity were determined on a NanoDrop spectrophotometer. RNA was treated with DNase I (amplification grade; Invitrogen) and reverse transcribed into cDNA by using random hexamers and Moloney murine leukemia virus reverse transcriptase (Invitrogen). mRNA levels for the genes of interest were determined with a CFX96 sequence detection system (Bio-Rad) with SYBR Green (Roche) as the fluorophore and gene-specific oligonucleotide primers. Primers used are as follows (forward, reverse): DC-SCRIPT (*ZNF366*): (5′-AAGCATGGAGTCATGGAG-3, 5′-TTCTGAGAGAGGTCAAAGG-3′), *PGK1*: (5′-CAAGAAGTATGCTGAGGCTGTCA-3, 5′-CAAATACCCCCACAGGACCAT-3′), *DUSP4*: (5′-AGTGGAAGATAACCACAAGG-3, 5′-GCTTAACGAACTCGAAGG-3′), *DUSP6*: (5′-GATCACTGGAGCCAAAAC-3, 5′-CAAGCAATGTACCAAGACAC-3′), *DUSP1*: (5′-AGTACCCCACTCTACGATCAGG-3, 5′-GAAGCGTGATACGCACTGC-3′), *DUSP5*: (5′-TGTCGTCCTCACCTCGCTA-3, 5′-GGGCTCTCTCACTCTCAATCTTC-3′), *DUSP10*: (5′-TTTGAAGAGGCTTTTGAGTT-3, 5′-GGGAGATAATTGGTCGTTT-3′). Reaction mixtures and program conditions were used as recommended by the manufacturer (Bio-Rad). Quantitative PCR data were analyzed with the CFX Manager software (Bio-Rad) and checked for correct amplification and dissociation of the products. mRNA levels of the genes of interest were normalized to mRNA levels of the housekeeping genes *GAPDH* or *PGK1* and were calculated according to the cycle threshold method (56).

### ELISA

Secreted IL-10 was measured using the human IL-10 ready-set-go kit (eBioscience) in the supernatants of 16–24 h-stimulated DCs or T cells. Secreted IFN-γ was measured using IFN-γ monoclonal antibodies; coating clone 2G1, detection Ab-biotin, clone XMG1.2 (both Thermo Fisher).

### Western Blotting

Monocyte-derived dendritic cells were lysed in a concentration of 10^6^/100 μL 4°C cold lysis buffer consisting of 62.5 mM Tris (pH 6.8, Sigma-Aldrich), 1% SDS (Invitrogen), and freshly added complete protease inhibitor cocktail (Roche), 1 mM PMSF (Sigma-Aldrich), and for phosphorylation-specific western blot: phosphatase inhibitors 1 mM Na3VO4 (Sigma-Aldrich) and 10 mM NaF (Merck). Cell lysates were mixed 1:4 with sample buffer containing 5% glycerol (Invitrogen), 6% SDS, 125 mM Tris–HCl (pH 6.8), 0.1 mg/mL bromophenol blue (Gebr. Schmid), and 10% 2-ME (Sigma-Aldrich), heated at 95°C for 5 min, and then cooled on ice. The proteins were resolved by electrophoresis on a 10% polyacrylamide gel (ratio of acrylamide to bisacrylamide, 37.5:1) and transferred to Protran nitrocellulose transfer membranes (Amersham) at 4°C. The following Abs were used for staining in TBS with 0.1% tween 20 (TBST) and 5% BSA (all from Cell Signaling Technology): rabbit anti-DUSP4 (1:1,000 dilution, clone: D9A5), rabbit anti-ERK (1:1,000 dilution, cat: 9102), rabbit anti-pERK (Thr202/Tyr204) (1:2,000 dilution, clone: D13.14.4E), rabbit anti-JNK (1:1,000 dilution, cat: 9252), rabbit anti-p38 (1:2,000 dilution, clone: D13E1), and rabbit anti-p-p38 (Thr180/Tyr182) (1:1,000 dilution, clone: D3F9). Goat anti-DC-SCRIPT were used for staining in PBS with 0.1% tween 20 and 1% skimmed milk powder (Campina) and 3% BSA (1:600 dilution, cat: AF4707, R&D Systems). Mouse anti-p-JNK (Thr183/Tyr185) were used for staining in TBST with 2% skimmed milk powder and 2% BSA (1:250 dilution, clone: G9, Cell Signaling Technology). Membranes were blocked for 1 h at room temperature, stained overnight with primary Abs [including rabbit anti-actin (1:2,000 dilution, clone: 20–33, Sigma-Aldrich) or rat anti-tubulin (1:2,000 dilution, clone: YOL1/34, Novus Biologicals)], and stained for 1 h at room temperature with corresponding secondary Abs (diluted 1:5,000): goat anti-mouse IgG IRDye 800CW, goat anti-rabbit IgG IRDye 800CW, donkey anti-goat IgG IRDye 800CW, donkey anti-rabbit IgG IRDye 680 (all Li-cor Biosciences), and goat anti-rat Alexa Fluor 680 (Invitrogen). Membranes were scanned by using an Odyssey Infrared Imaging System (Li-cor Biosciences).

### Flow Cytometry of DCs

For phosphorylation-specific flow cytometry, paraformaldehyde (Merck) was added directly to the culture medium at a final concentration of 1.6% at the end of the stimulation. Cells were fixed for 10 min at room temperature. Subsequently, culture medium was aspirated, resuspended in 100% ice-cold MeOH (Boom) and incubated at −20°C overnight, followed by extensive washing (4×) in PBS with 1% BSA, 0.05% NaN3 (Merck). Cells were blocked and stained in PBS with 1% BSA, 0.05% NaN3 (Merck), and 2% human serum (Sanquin), using 1:200 diluted pERK, 1:400 diluted p-p38 Abs, or isotype control (rabbit IgG, Jackson Immuno Research). Cells were stained with primary Ab/isotype control for 45 min on ice, followed by 30 min on ice with a secondary PE-Cy5.5 goat anti-rabbit IgG Ab (Invitrogen, cat# L42018). Data were acquired on an FACSCyan (Beckman Coulter). Isotype controls gave a staining intensity similar as unstimulated moDCs, indicating that blocking conditions were sufficient to avoid unspecific staining (data not shown). Viability was measured using a fixable viability dye eFluor780 (eBioscience), following the manufacturers instructions. Data were analyzed using FlowJo (Treestar).

### Chromatin Immunoprecipitation Sequencing

Chromatin immunoprecipitation was performed as previously described (31), using 20 µg goat-α-DC-SCRIPT Ab (R&D Systems). 10 ng of input or ChIP-enriched DNA was end-paired using T4 DNA polymerase, *E. coli* DNA Pol I large fragment (Klenow polymerase, New England Biolabs), and T4 polynucleotide kinase (New England Biolabs), followed by purification using the QIAquick PCR purification kit (Qiagen). Subsequently, DNA was dA-tailed using the Klenow fragment (3′ to 5′ exo minus, New England Biolabs), followed by purification using the MinElute Reaction Cleanup kit (Qiagen). Next, DNA was ligated to multiplex NEXTflex adapters (Bioo Scientific). IP and input DNA were purified by the MinElute reaction Cleanup kit, and amplified by PCR using the KAPA HiFi HotStart ReadyMix PCR kit (KAPA Biosystems) with the following program: 45 s at 98°C for initial denaturation, 15 s at 98°C, 30 s at 65°C, 30 s at 72°C for four cycles, followed by 1 min at 72°C for final extension. Removal of excess adaptors and selection of 300 bp bands was done using 2% E-Gel SizeSelect Agarose Gels (Invitrogen). Adapter-modified DNA fragments were enriched by PCR using the KAPA HiFi HotStart ReadyMix PCR kit for 8–10 cycles with the aforementioned program. To get rid of the 120 bp adapter dimer, the PCR product was purified using Ampure beads (Beckman Coulter). Libraries were sequenced on the Illumina HiSeq 2000. H3K4me3 and H3K27ac antibodies were extensively characterized^1^ and used for ChIP according to standard BLUEPRINT protocols.^1^

### ChIP-Seq Data Processing

Peaks were called using the algorithm MACS2 version 2.0.10.20120913 (57) with default settings. In order to account for donor variation and dynamics, three donors were assayed, using immature, 1 h-, and 24 h-stimulated DCs. Peaks present in all three donors were determined using intersect with BEDTools version 2.20.1 (58), and only DC-SCRIPT peaks present in all three donors were used for subsequent analysis. All ChIP-Seq data have been submitted to the GEO database (accession number: GSE78923) *k*-Means clustering (*k* = 6, Euclidean distance) and heatmaps were generated using Fluff (59). *De novo* motif analysis was done using GimmeMotifs version 0.8.6 (34). The motif was trimmed to remove low information content containing bases.

### Cyclic Amplification and Selection of Targets

Human DC-SCRIPT was cloned in the pCATCH vector (60), as BamHI-XbaI inserts. *In vitro* transcription/translation was performed with the TNT T7 Quick Coupled Transcription/Translation System (Promega) according to the manufacturer’s recommendations, using 1 µg of DNA as input. Transcription/translation took place at 30°C for 90 min. 10% of the reaction was tested by western blot analysis to verify protein production, while 20% was used in each CAST round.

Oligo-nucleotides carrying defined ends and a 21-nt region of degeneracy (5′-GCCTCCATGGACGAATTCTGT-(N)_21_-AGCGGATCCCGCATATGACCG-3′) and PCR primers (forward: 5′- GCCTCCATGGACGAATTCTGT-3′ and reverse: 5′-CGGTCATATGCGGGATCCGCT-3′) were used during CAST. As a first step, double-stranded oligo-nucleotides were prepared as follows. 8.5 µg of the degenerative nucleotides were mixed with 4.3 µg of the reverse primer in 50 µL of Tris–HCl (100 mM, pH 8) and heated at 80°C, then cooled down slowly to 4°C. 2 µL of the hybridized oligo-nucleotides were used together with 2 U of the Klenow fragment of DNA polymerase I (37°C for 1 h) to create dsDNA. dsDNA was precipitated and used in the first round of CAST. Each CAST round was performed in binding buffer containing 30 mM HEPES pH 7.4, 100 mM NaCl, 0.01% NP40, 0.01 mg/mL BSA, 0.05 mM ZnSO_4_, 2 mM MgCl_2_, 0.6 mM PMSF, and 10% glycerol. In brief, 500 µL of binding buffer were mixed with 20% *in vitro* transcribed/translated proteins and DNA and incubated for 30 min at 4°C. Then 10 µL of Protein G beads and 3 µg of mouse M2 anti-FLAG mAb (Sigma) were added and incubated overnight. Precipitated dsDNA was used for the first round of CAST, or 80% of the PCR reaction for the subsequent rounds. After the binding reaction, Protein G beads were washed twice with 600 µL of binding buffer and resuspended in 20 µL of 5 mM EDTA pH 8 for 10 min at 90°C. Beads were pelleted and supernatant was used for PCR. 20% of the PCR reaction was tested on gel to verify DNA precipitation and amplification by CAST.

PCR reactions for CAST were performed using 100 ng of forward and reverse primer, 0.5 mM of dNTPs, 5 mM of MgCl_2_, and 2.5 U of Taq polymerase with 58°C as an annealing temperature. The number of PCR cycles was kept to a minimum, i.e., 15 cycles, in the first two rounds. Minimal PCR amplification helped to reduce the amplification of non-specific oligo-nucleotides and the formation of hetero-duplexes that resulted from the re-annealing of products that were mismatched in the 21-bp central-region. After the third round, however, 20 cycles of PCR ensured good amplification and abundance of specific oligo-nucleotides. Sequences from four rounds of CAST were used as input for MEME (35) to generate a consensus sequence for DC-SCRIPT.

### Genomic Regions Enrichment of Annotations Tool

Genomic Regions Enrichment of Annotations Tool analysis was done in version 3.0.0 as previously described (36), with DC-SCRIPT binding sites with an H3K4me3 and/or H3K27ac histone mark, and containing the GA-rich motif. Default settings were employed, i.e., basal plus extension: proximal: 5.0 kb upstream, 1.0 kb downstream, plus distal: up to 1,000.0 kb. Statistical significance is based on false discovery rate (cutoff: 0.05). Displayed data contains minimum three genes in each GO.

### Luciferase Assays

The DNA sequence underlying the ChIP-Seq-identified DUSP4 EA-SC-binding site was cloned into a pGL4.10 luciferase vector (Promega) behind a DUSP4 promoter. For comparison, a control pGL4.10 vector with the DUSP4 promoter and a piece of genomic DNA of similar size, and located between the DUSP4 TSS and the DUSP4 EA-SC binding site was also generated. These vectors were transfected into HEK293 [ATCC, tested to be mycoplasma free using mycoalert mycoplasma detection kit (Lonza), and used between passages 2–15 after thawing] together with a renilla control vector (pRL-TK, Promega) and increasing amounts of a pCATCH-DC-SCRIPT expression vector. HEK293s were plated 24 h before transfection using metafectene. Cells were harvested after 24 h, and cell lysates were analyzed for luminescence according to the manufacturer’s protocol (Dual Luciferase Reporter assay, Promega) using a Victor3 luminometer (PerkinElmer). Relative light units were calculated after correction for transfection efficiency based on the activity of the cotransfected pRL-TK.

### Overexpression of DUSP4

To generate a DUSP4/GFP expression vector, DUSP4 (NM_001394) was cloned into the expression vector pEGFP-N3 (Clontech, Mountain View, CA, USA), using the restriction enzyme sites BglII and BamHI. As a control, the empty pEGFP-N3 vector was used. Immature SC-KD-DCs and Ctrl-DCs were harvested on day 6 and electroporated using the Neon transfection system (Invitrogen) according to the manufacturer’s instructions. Briefly, 10^6^ DCs were mixed with 5 µg DNA, and electroporated with two pulses of 1,000 V for 40 ms. Subsequently, DCs were seeded in microtiter plates and rested for 5–6 h until GFP was visible. The cells were subsequently stimulated and used for functional assays.

### Naïve T Cell Polarization

Naïve T cells were isolated from buffy coats using magnetic-associated cell sorting, and negative selection, by depleting cells expressing CD8a, CD14, CD15, CD16, CD19, CD36, CD56, CD132, TcRγ/δ, and CD235a (CD4+ T Cell isolation kit, human, Miltenyi), and CD45RO [anti-CD45RO-PE (DAKO) plus anti-PE microbeads (Miltenyi)]. Purity was checked using CD3-FITC (BD), CD4-PE-Cy7 (BioLegend), CD45RA-APC-Cy7 (BioLegend), and CD45RO-PE and determined to be >97% of CD3+ CD4+ CD45RO− CD45RA+ T cells.

SC-KD-DCs or Ctrl-DCs were stimulated for 16 h with R848, washed and counted, before co-culturing with naïve CD4+ T cells in a ratio of 5,000:20,000 DC:T. The super antigen SEB (Sigma-Aldrich) was added at 10 pg/mL. After 5, 7, and 9 days of co-culture, the T cells were split 1:2 and recombinant human IL-2 were added at a final concentration of 20 U/mL. On day 11, the T cells are in a resting state, which can be seen in a light microscope by the T cell clusters falling apart and cells are rounded. The resting T cells were assayed for intracellular transcription factor expression using the cytofix/cytoperm kit (BD). Prior to permeabilization cells were stained with fixable viability dye eFluor780 (eBioscience). Antibodies used for staining were: anti-human Gata-3-Alexa Fluor488, anti-human RORgamma(t)-APC, and anti-human T-bet-PE (all eBioscience).

For cytokine production, T cells were harvested, counted, and 100,000 cells re-plated in a 96-well round bottom plate, followed by addition of 100,000 anti CD3/CD28 beads (Gibco). Supernatant was harvested after 24 h and assayed by ELISA. Data presented in the figure consists of two T cell donors and six DC donors.

## Availability of Data

The datasets generated during the current study are available in the GEO repository, under accession number: GSE78923.

## Ethics Statement

Buffy coats were obtained from healthy volunteers (Sanquin, Nijmegen, The Netherlands) after informed consent in accordance with the Declaration of Helsinki. The study was approved by the Institutional Review Board of the Radboud University Nijmegen Medical Center, Commissie Mensgebonden Onderzoek.

## Author Contributions

Conceptualization: JS, MA, and GA; methodology: JS, SH, MA, and GA; software: SH; formal analysis: JS and SH; investigation: JS, ML, CT, VT, PL, EJ-M, AS, and MA; writing—original draft: JS, SH, MA, and GA; writing—review and editing: JS, SH, ML, CT, VT, PL, EJ-M, AS, JM, CL, HS, MA, and GA; visualization: JS and SH; funding acquisition: JS, CT, JM, MA, and GA.

## Conflict of Interest Statement

The authors declare that the research was conducted in the absence of any commercial or financial relationships that could be construed as a potential conflict of interest.

## Footnotes

^1^www.blueprint-epigenome.eu (Accessed: April 18, 2018).

## Supplementary Material

Table S1 GREAT analysis, GO Biological Process.

Table S2 GREAT analysis, GO Molecular Function.
