# Supplementary material for: Dendritic Cells Actively Limit Interleukin-10 Production Under Inflammatory Conditions via DC-SCRIPT and Dual-Specificity Phosphatase 4
Source: Front Immunol. 2018 Jun 22;9:1420. doi: 10.3389/fimmu.2018.01420 (PMC6023963; doi:10.3389/fimmu.2018.01420)
Supplement: Supplementary file 3 [file image_1.PDF]

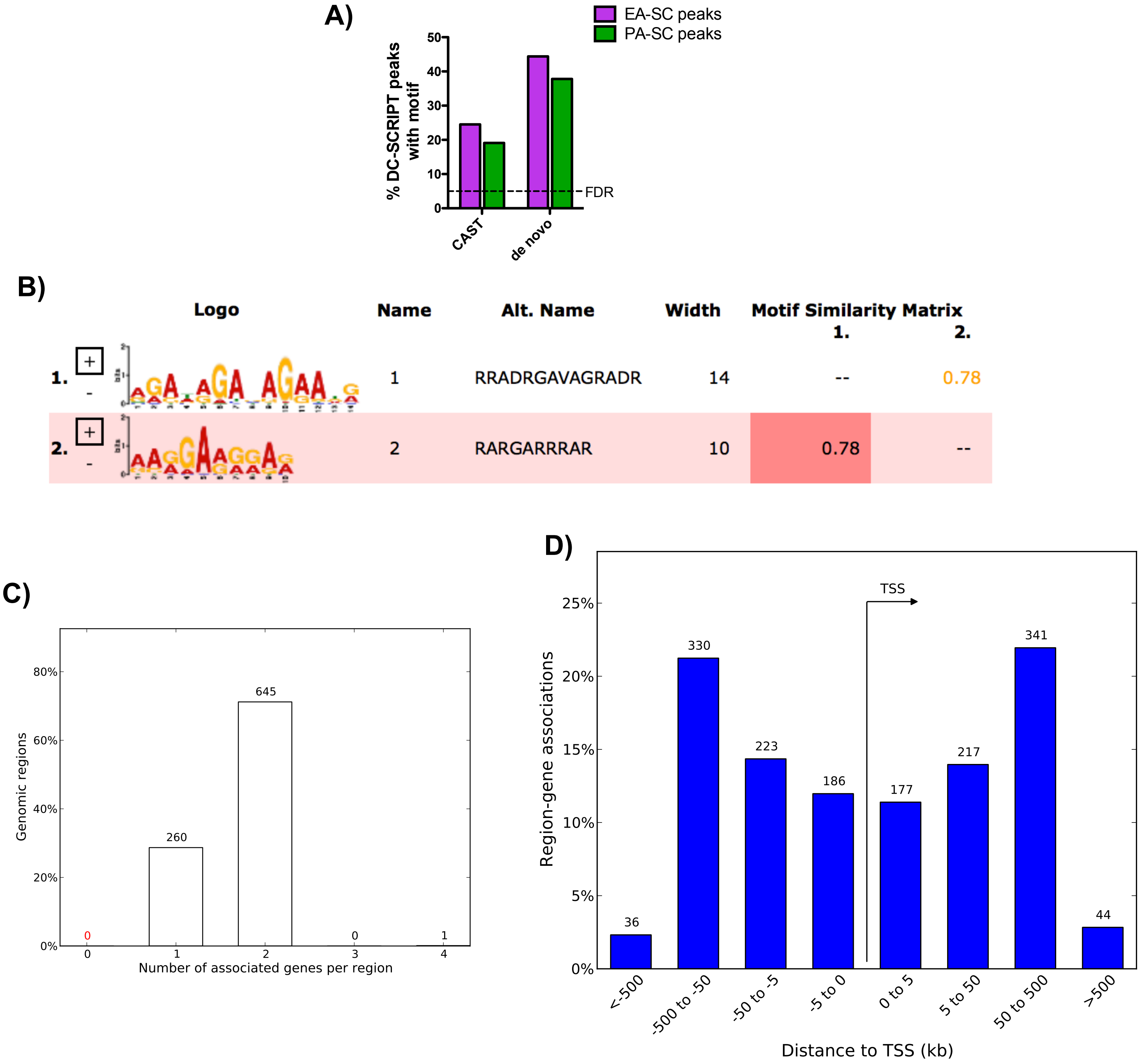

Figure S1, related to Fig. 1: DC-SCRIPT binds GA-rich sequences. (A) Percentage of EA-SC (purple) and PA-SC (green) binding sites containing the CAST and the *de novo* motif (FDR = 5%). (B) Similarity between the *in vitro* CAST motif (1) and the *de novo* motif (2) calculated by pairwise comparisons using Motif-based sequence analysis tool (MAST) from the MEME suite. The *de novo* motif is marked with a pale red background because MAST recommends not to use it for motif scanning due to high similarity to the *in vitro* motif. (C-D) GREAT analysis was conducted with DC-SCRIPT peaks containing the GA-rich DNA motif. (C) Number of associated genes per region. (D) Distance of assayed DC-SCRIPT peaks to transcription start site (TSS) of genes.
