# Supplementary material for: Dendritic Cells Actively Limit Interleukin-10 Production Under Inflammatory Conditions via DC-SCRIPT and Dual-Specificity Phosphatase 4
Source: Front Immunol. 2018 Jun 22;9:1420. doi: 10.3389/fimmu.2018.01420 (PMC6023963; doi:10.3389/fimmu.2018.01420)
Supplement: Supplementary file 4 [file image_2.PDF]

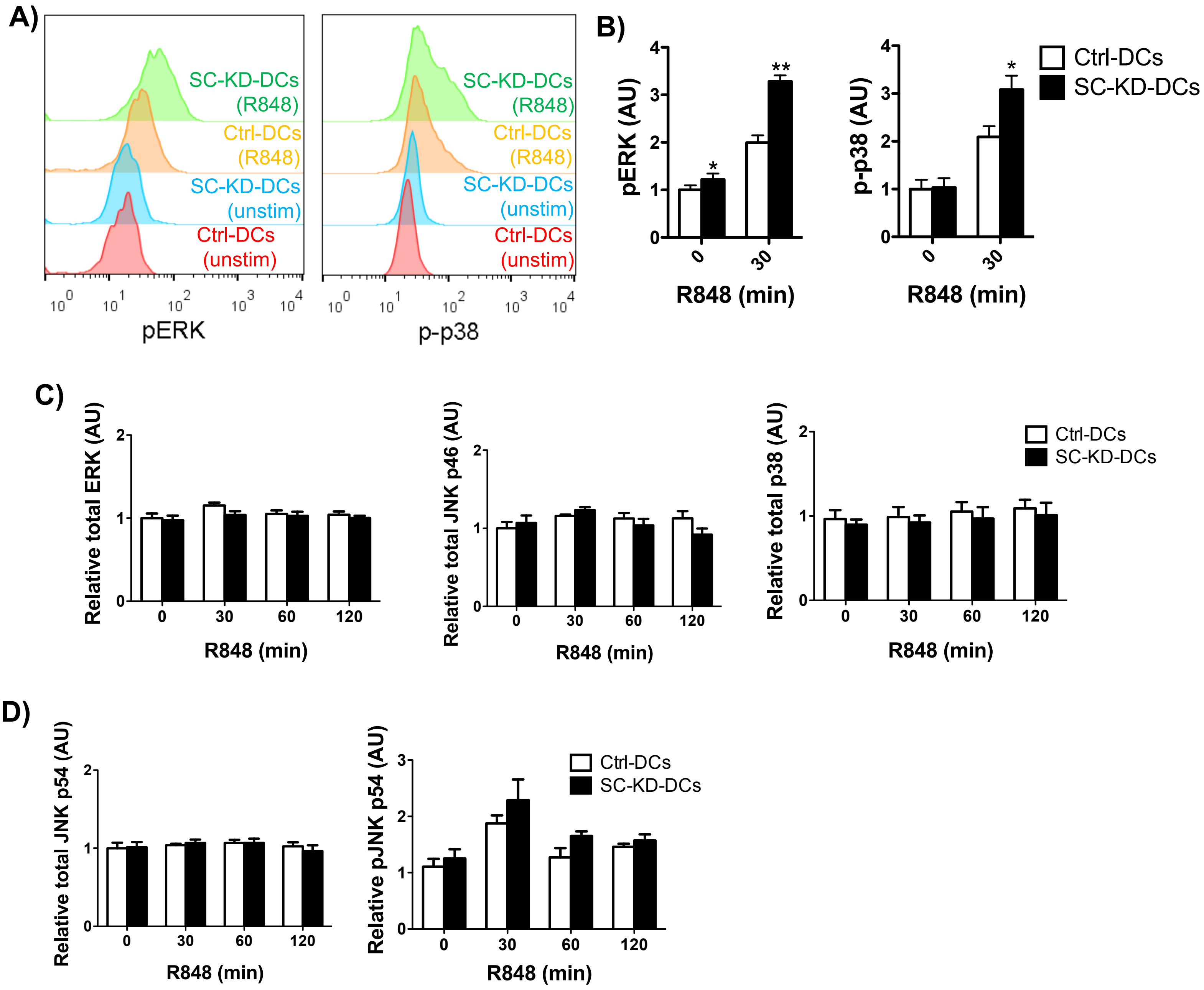

Figure S2, related to Fig. 2: Phosphoflow and total protein/mRNA quantification. Human monocyte-derived dendritic cells (moDCs) were treated with siRNA for DC-SCRIPT (SC-KD-DCs) or non-targeting siRNA as a control (Ctrl-DCs) during differentiation into DCs. (A-B) Ctrl-DCs and SC-KD-DCs were stimulated with R848 (TLR7/8 ligand) for 30 min, and assayed for phosphorylation of the 3 major MAPKs: ERK, JNK, and p38. The pJNK Ab did not work in FACS, and data are not displayed. (A) Representative FACS histograms of pERK and p-p38. (B) Quantification of relative mean fluorescent intensity (n = 5-8, mean +SEM). (C-D) Ctrl-DCs and SC-KD-DCs were stimulated with R848 for 0-120 min, and assayed for total expression of the 3 major MAPKs: ERK, JNK, and p38. JNK was divided into p46 and p54 isoforms. Phosphorylated p-p54 is also displayed. n = 5, mean +SEM. Statistics: student's paired two-tailed t test, * $p < 0.05$. ** $p < 0.01$.
