# Supplementary material for: Dendritic Cells Actively Limit Interleukin-10 Production Under Inflammatory Conditions via DC-SCRIPT and Dual-Specificity Phosphatase 4
Source: Front Immunol. 2018 Jun 22;9:1420. doi: 10.3389/fimmu.2018.01420 (PMC6023963; doi:10.3389/fimmu.2018.01420)
Supplement: Supplementary file 5 [file image_3.PDF]

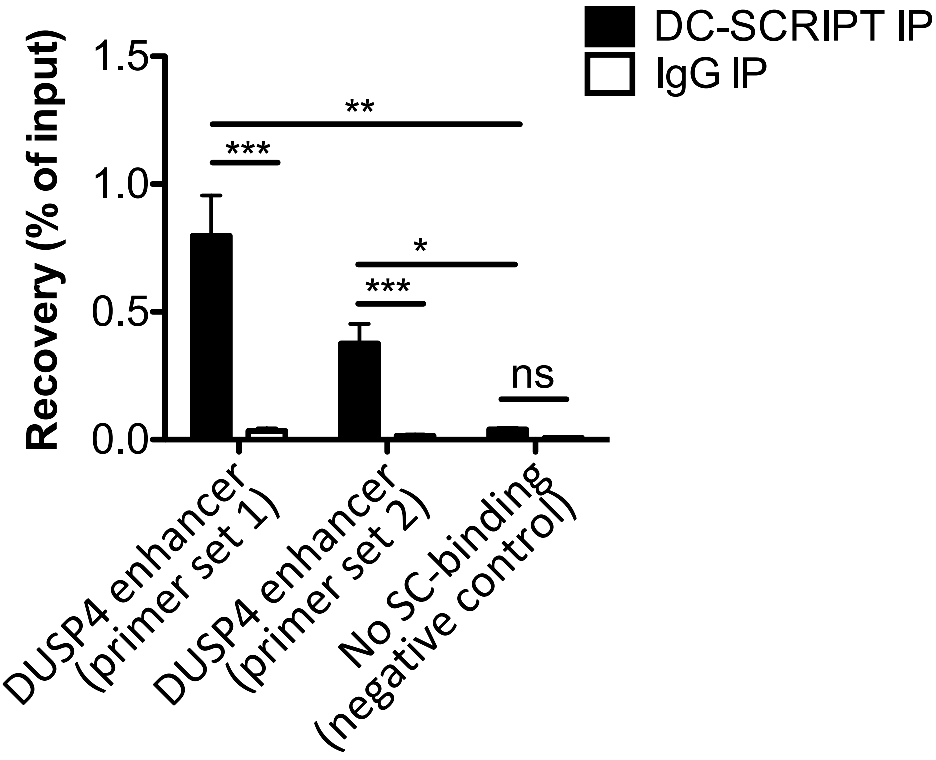

Figure S3, related to Fig. 3: ChiP-PCR validation of DC-SCRIPT binding to DUSP4 enhancer. Immature DCs were ChIPed with a DC-SCRIPT Ab or isotype control (IgG). The ChIPed product and input chromatin for the ChIP was assayed for DUSP4 enhancer binding by qPCR, using 2 sets of primers specific for the DUSP4 enhancer. As a negative control a primer set specific for a genomic location between the DUSP4 gene and the DUSP4 enhancer without any detected DC-SCRIPT binding (by ChiP-seq) was used. The recovery was calculated by relating the ChIPed product to the input. Statistics: ANOVA with a Bonferroni posttest; $n = 7$; error bars = SEM. *$p < 0.05$, **$p < 0.01$, ***$p < 0.001$.

Primers used are as follows (forward, reverse): primer set 1 (chr8:29492412-29492529): (5'-AATTTACCAAGAGCACGTTT-3, 5'-CATTCTCTCTCTCCTTCTGG-3'), primer set 2 (chr8:29492511-29492621): (5'-CAGAAGGAGAGAGAGAATGG-3, 5'-ATTGCATGAAGCTGAAGAA-3'), negative control (chr8:29324042-29324152): (5'-AAAATGGAAATACGGAGAGA-3, 5'-GGCTTAATGGAAGTGAGAAA-3').
