# Supplementary material for: Dendritic Cells Actively Limit Interleukin-10 Production Under Inflammatory Conditions via DC-SCRIPT and Dual-Specificity Phosphatase 4
Source: Front Immunol. 2018 Jun 22;9:1420. doi: 10.3389/fimmu.2018.01420 (PMC6023963; doi:10.3389/fimmu.2018.01420)
Supplement: Supplementary file 6 [file image_4.PDF]

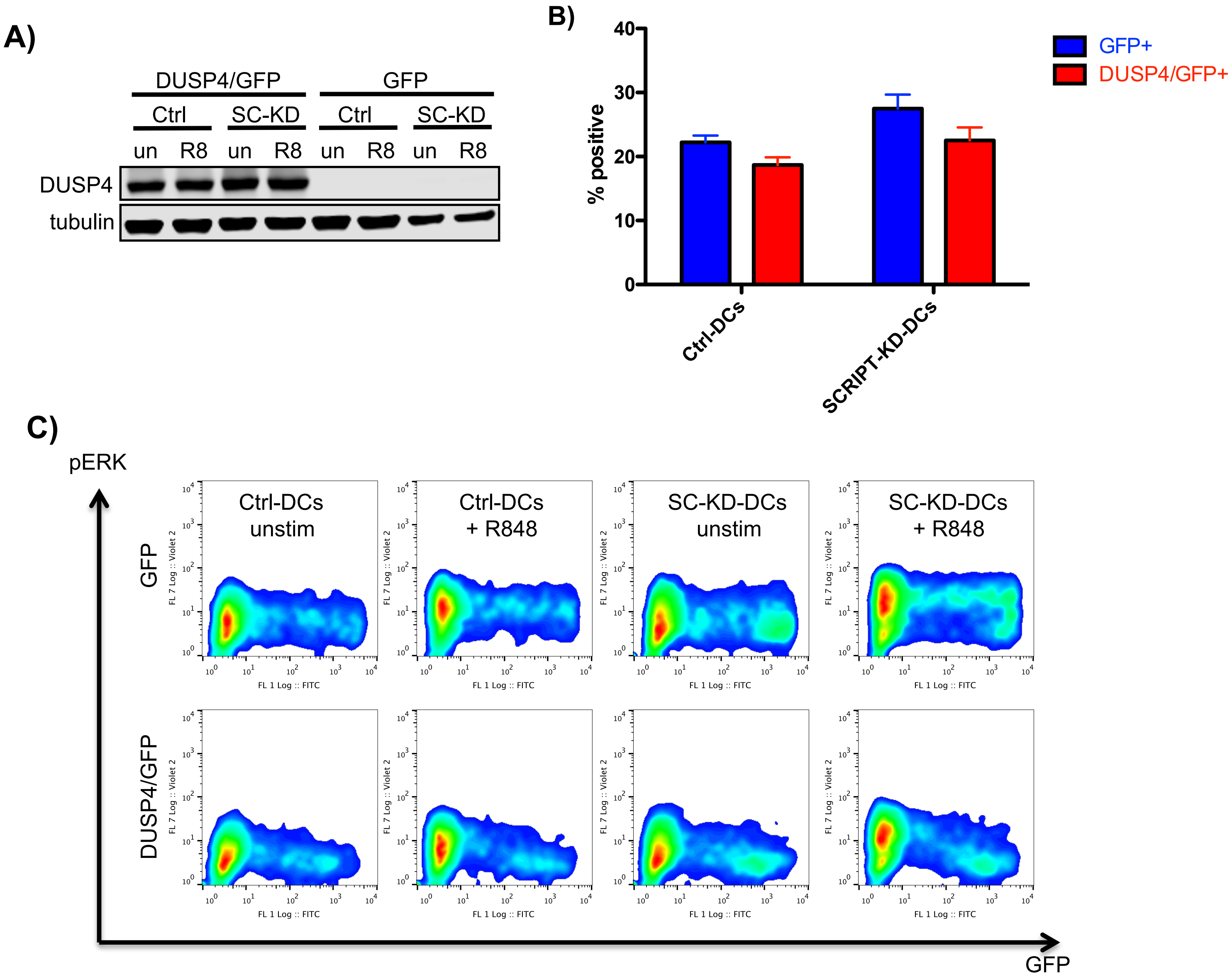

Figure S4, related to Fig. 4: Rescuing DUSP4 expression diminished DC-SCRIPT-knockdown-mediated ERK phosphorylation. Immature Ctrl-DCs and SC-KD-DCs were transfected with a DUSP4/GFP or a GFP control vector. (A) Representative WB of DUSP4/GFP overexpression (unstimulated (un), R848 stimulated (R8)). Six hours after transfection, cells were stimulated for 30 min with R848 and assayed by FACS for (B) transfection efficiency (n = 8, mean + SEM) and (C) pERK levels. (C) is representative for 8 donors summarized in Fig. 4 of the paper.
